# Algoritmo Simplificado para o Diagnóstico Diferencial das Taquicardias com QRS Largo. Seria Essa a Melhor Forma de Treinar os Jovens Médicos?

**DOI:** 10.36660/abc.20201344

**Published:** 2021-03-03

**Affiliations:** 1 Serviço de Arritmias do Hospital do Coração de São Paulo São PauloSP Brasil Serviço de Arritmias do Hospital do Coração de São Paulo, São Paulo, SP - Brasil.

**Keywords:** Taquicardia Ventricular, Emergências, Primeiros Socorros, Algoritmos, Diagnóstico, Unidade de Terapia Intensiva

Fazer o diagnóstico correto de uma taquicardia ventricular em uma sala de emergência (SE), com pouco tempo de decisão e somente com o eletrocardiograma (ECG) é um desafio que depende de muito preparo por parte do médico que atende esse paciente. Qualquer instrumento que venha a facilitar e agilizar esse processo, principalmente aumentando o grau de acurácia, é também digno de elogios, principalmente considerando que, nem sempre, o médico plantonista é cardiologista, ou menos provável, é um arritmologista treinado o suficiente para fazer esse diagnóstico com precisão.

Uma taquicardia ventricular, uma taquicardia supraventricular (em um paciente com distúrbio de condução prévio ou com aberrância de condução) e uma taquicardia supraventricular conduzida por um feixe anômalo podem apresentar traçados eletrocardiográficos semelhantes e, por vezes, muito difíceis de diferenciar, tornando a decisão médica um risco para a vida do paciente. Para que essa diferenciação seja possível, foram criados vários algoritmos que utilizam sinais eletrocardiográficos para auxiliar e permitir o diagnóstico correto e, consequentemente, o tratamento adequado, como é o caso do algoritmo de Brugada,^1^ largamente conhecido (e que, na verdade, é uma combinação de critérios de Brugada e de Wellens^2^), o de Vereckei^3^ e o de Pava,^4^ entre outros. Todos os algoritmos criados apresentam sensibilidade e especificidade (S/E) variáveis que os tornam mais precisos ou menos; no entanto, na mesma proporção, esses algoritmos podem ser mais simples ou mais complexos (que dependem de maior treinamento do operador e maior tempo para chegar ao diagnóstico).

Os autores do artigo “Validation of a Simple ECG Algorithm to Recognize Ventricular Tachycardia”^5^ buscam uma ferramenta que permita que os médicos com menos treinamento cheguem ao diagnóstico diferencial de uma forma mais simples e em menor tempo, utilizando um algoritmo bastante simplificado, desenvolvido inicialmente por Nagi et al.,^6^ mas que não foi adequadamente validado, conforme explicação desses autores. A proposta foi validar o algoritmo comparando com grupos de examinadores mais experientes *versus* menos experientes e os ECG sem história clínica e com história clínica. O diagnóstico preciso da taquicardia foi avaliado previamente pelo estudo eletrofisiológico. Um número expressivo de avaliações resultou dessa combinação, o que permitiu a análise estatística de E/S confrontado com o algoritmo de Brugada. A análise final mostrou que o novo algoritmo foi semelhante ao de Brugada, com menor índice de conflito entre os examinadores, mostrando maior reprodutibilidade. A discussão fica exatamente nas limitações, pois o algoritmo foi validado somente em pacientes que não estavam utilizando fármacos antiarrítmicos e também não foram incluídos casos com pré-excitação ventricular. Ambas as condições não podem ser descartadas entre os pacientes que chegam à SE em taquicardia com QRS largo. Também é temerário considerar taquicardia supraventricular todos os casos que não cumprirem os quatro critérios desse algoritmo. Em minha experiência, o melhor é confrontarmos os vários algoritmos para reforçar a suspeita diagnóstica, fato já publicado por outros autores.^7^ Além disso, há que se considerar a simplicidade do algoritmo de Vereckei, já extensamente validado e facilmente memorizado pelos menos experientes. Outra limitação é a necessidade do ECG completo, condição que nem sempre está presente nos monitores da SE e da unidade de terapia intensiva (UTI). Nesse caso, um novo e simples algoritmo foi idealizado,^8^ permitindo boa E/S com os algoritmos mais complexos. Infelizmente, não há um algoritmo que permita 100% de E/S, e acredita-se que teremos que conviver com essas discussões ainda por um longo tempo. No entanto, ao utilizar os critérios clínicos associados ao algoritmo dominado pelo médico avaliador, podemos auxiliar ao máximo os pacientes que se encontram nessas situações críticas.
