# Supplementary material for: Algoritmo Simplificado para o Diagnóstico Diferencial das Taquicardias com QRS Largo. Seria Essa a Melhor Forma de Treinar os Jovens Médicos?
Source: Arq Bras Cardiol. 2021 Mar 3;116(3):464–5. [Article in Portuguese] doi: 10.36660/abc.20201344 (PMC8159567; doi:10.36660/abc.20201344)
Supplement: Translation

# Translation: Simplified Algorithm for Differential Diagnosis of Tachycardias with Wide QRS. Is this the Best Way to Train Young Doctors?

**Affiliations:** 1 São PauloSP Brazil Serviço de Arritmias do Hospital do Coração de São Paulo, São Paulo, SP - Brazil.

**Keywords:** Tachycardia, Emergencies, First Aid, Algorithms, Diagnosis, Algorithms

To make the correct diagnosis of ventricular tachycardia in an emergency room (ER), with little time for decision-making and based only on the electrocardiogram is a challenge that depends on a lot of preparation by the doctor. Any instrument that facilitates and streamlines this process, especially increasing the degree of accuracy, is also worthy of praise, especially considering that the physician on duty is not always a cardiologist, or even less likely an arrhythmologist, sufficiently trained to make an accurate diagnosis.

An episode of ventricular tachycardia, supraventricular tachycardia (in a patient with a previous conduction disorder or with a conduction aberrancy) and supraventricular tachycardia caused by anomalous bundles may show similar electrocardiographic tracings, which are sometimes very difficult to differentiate, making the medical decision a risk to the patient’s life. To make this differentiation possible, several algorithms have been created that use electrocardiographic signals to assist and allow the correct diagnosis to be made and, consequently, to establish the adequate treatment, as in the case of the Brugada algorithm which is widely known (and that in fact is a combination of Brugada and Wellens criteria), as well as Vereckei and Pava criteria among others. All the created algorithms have sensitivity and specificity (S/E) variables that make them more or less accurate; however, in the same proportion, these algorithms can be simpler, or more complex (depending on the operator’s better training and greater time to reach the diagnosis).

The authors of the article “Validation of a Simple Electrocardiographic Algorithm for Detection of Ventricular Tachycardia” aim at obtaining a tool that allows physicians with less training to reach a differential diagnosis in a simpler way and in less time, using a very simplified algorithm, initially developed by Nagi et al. but that was not properly validated, as explained by these authors. The proposal was to validate the algorithm by comparing more experienced *versus* less experienced groups of examiners and ECGs with no clinical history and with clinical history. The precise diagnosis of tachycardia was previously evaluated through the electrophysiological study. A significant number of evaluations resulted from this combination, which allowed the statistical analysis of S/E compared with the Brugada algorithm. The final analysis showed that the new algorithm was similar to Brugada’s, with a lower rate of conflict between the examiners, showing greater reproducibility. The discussion is precisely in the limitations of the study, because the algorithm was validated only in patients who were not using antiarrhythmic drugs and cases with ventricular pre-excitation were not included either. Both conditions cannot be ruled out in patients who arrive at the ER with tachycardia and a wide QRS interval. It is also careless to consider supraventricular tachycardia in all cases that do not meet the four criteria of this algorithm. In my experience, it is best to confront the various algorithms to reinforce the diagnostic suspicion, a fact that has already been discussed by other authors and we must also consider the simplicity of the Vereckei’s algorithm, which has been widely validated and is easily memorized by the least experienced doctors.

Another limitation is the need for a complete ECG, a test that is not always available in ERs and IUC monitors. In this case, a new and simple algorithm was idealized, allowing good S/E in relation to the most complex algorithms. Unfortunately, there is no algorithm that allows 100% S/E, and I believe we will have to live with these discussions for a long time yet, but, using the clinical criteria associated with the algorithm mastered by the evaluating physician, we can help as much as possible the patients who find themselves in these critical situations.
